# High intensity interval training alters gene expression linked to mitochondrial biogenesis and dynamics in high fat diet fed rats

**DOI:** 10.1038/s41598-025-86767-5

**Published:** 2025-02-14

**Authors:** Mohammad Jahangiri, Shahnaz Shahrbanian, Reza Gharakhanlou

**Affiliations:** Department of Sport Science, Faculty of Humanities, Tarbiat Modares University, Tehran, Iran

**Keywords:** Obesity, Exercise Training, Mitochondrial Biogenesis, Mitochondrial dynamic, Fusion, Fission, Cell biology, Physiology, Metabolism, Endocrinology, Endocrine system and metabolic diseases, RNA

## Abstract

A High-Fat Diet (HFD) leads to disruption of mitochondrial biogenesis and dynamics. Exercise training, especially High-Intensity Interval Training (HIIT) increases mitochondrial biogenesis and dynamics. The present study aimed to investigate the effect of a period of HIIT with and without HFD consumption on the expression of peroxisome proliferator-activated receptor gamma coactivator 1-alpha (Pgc1-α), Mitofusins-2 (Mfn2), Optic atrophy-1 (Opa1), Dynamin-related protein-1 (Drp1) and mitochondrial Fission protein-1 (Fis1) genes as indicators of mitochondrial biogenesis and dynamics function in the soleus muscle of male Wistar rats. Twenty-four healthy male Wistar rats were randomly divided into four groups: (1) Control, (2) Control + HIIT, (3) HFD, and (4) HFD + HIIT. The HIIT training protocol lasted for 10 weeks with a frequency of 3 sessions per week. The Real-Time Quantitative Reverse Transcription PCR method was used to investigate the gene expression. One-way ANOVA and Fisher’s post-hoc analyses were used to examine group differences. HFD consumption caused an increase in weight (*P* < 0.05), the expression of Drp1 and Fis1 genes (*P* < 0.001), and a decreased expression of Pgc1-α, Mfn2, and Opa1 genes (*P* < 0.001). HIIT training increased the expression of PGC1-α (*P* = 0.009), Mfn2 (*P* < 0.004), and Opa1 (*P* < 0.011) genes, while it decreased the expression of Drp1 (*P* = 0.003) and Fis1 genes (*P* = 0.027). These findings suggest that HIIT can counteract the negative effects of HFD on mitochondrial function by modulating gene expression related to mitochondrial biogenesis and dynamics.

## Introduction

Obesity is a complex multifactorial global disease and its prevalence has rapidly increased worldwide over the past 40 years^1^. Obesity has a negative effect on all physiological functions of the body, including mitochondrial health and function, and leads to disruption of mitochondrial biogenesis and dynamics^2^. Mitochondrial health is an essential mediator of cellular function in various tissues. Mitochondria are highly dynamic organelles that constantly undergo a coordinated cycle of biogenesis, fusion, fission, and destruction, forming a complex network to respond to dynamic changes in energy requirements^3^. Evidence suggests that mitochondrial dysfunction is often modulated by genetic, environmental, and lifestyle factors^4^. For obesity, researchers state many predisposing factors such as geographic region, social conditions, political, economic and genetic factors that have different effects on the prevalence of obesity and overweight. Also, the most common demographic factors are behavioral factors (food habits and lifestyle), genetic factors, and living in developed and industrial areas with high machine facilities^5^. Lifestyle changes in recent decades, including inactivity or reduced physical activity and consumption of high-calorie foods including high-fat diet (HFD), are the most important and common causes of obesity^6,7^ and disruption of mitochondrial biogenesis and dynamics^8,9^.

Several studies have shown that HFD decreases peroxisome proliferator-activated receptor gamma coactivator 1-alpha (Pgc1-α) levels and ultimately decreases mitochondrial biogenesis in gastrocnemius^10^ and cardiac muscles^11^. On the other hand, some studies have also shown that the consumption of HFD increases the levels of Pgc1-α in the Extensor Digitorum Longus (EDL)^12^, quadriceps^13^, and soleus^14^ muscles. Mitochondrial dynamics are regulated by fusion and fission processes^15^. Fusion is carried out by Mitofusins-1, 2 (Mfn1, Mfn2), and Optic atrophy-1 (Opa1)^15^. Dynamin-related protein-1 (Drp1) and mitochondrial Fission protein-1 (Fis1) are responsible for mitochondrial fission^15^. Studies have demonstrated that HFD disrupts the balance of mitochondrial dynamics in skeletal muscle^16,17^, which may be linked to various disorders and diseases such as obesity, diabetes^18^, cardiomyopathy, ischemia, heart infarction, and neurodegenerative conditions like Huntington’s, Parkinson’s, and Alzheimer’s^15^.

Exercise training may prevent mitochondrial dysfunction by improving mitochondrial biogenesis^19–21^, dynamics^22^ quality control and metabolism^23^. Also, various types of exercise such as resistance^24–26^, endurance^27^ and High-Intensity Interval Training (HIIT)^28^ have been shown to increase the expression of Pgc1-α in various tissues including the gastrocnemius^25,26^, soleus^28^ and plantaris^24^ muscles and improve mitochondrial biogenesis. Other research showed that HIIT training improves mitochondrial dynamics^6,29^.

Several studies have investigated the effect of exercise training after a period of consuming an HFD on mitochondrial biogenesis and dynamics in different animal tissues, including the liver^6,30^, brain (hippocampus)^31^, gastrocnemius muscle^32,33^ and EDL muscle^12^. One study was done on the soleus muscle^29^. After 12 weeks of HFD to induce diabetes, Peyravi et al.^29^ investigated the effect of 8 weeks of HIIT training on the expression of mitochondrial dynamic genes in the soleus muscle of male Sprague Dawley rats. In most of the studies, the rats were first fed with HFD, and after the development of disease conditions such as obesity and diabetes, exercise interventions were applied^6,29,33^. In these studies, exercise training has been used as a non-pharmacological treatment method to reduce the consequences of diseases such as obesity and diabetes. According to our knowledge, no study has investigated the simultaneous effects of HIIT training and HFD consumption on the expression of genes related to mitochondrial biogenesis and dynamics in healthy male rats. Hence, we hypothesize that HIIT training as a preventive modality moderates the consequences of HFD consumption. Therefore, the present study aimed to investigate the effect of HIIT training on the expression of genes related to mitochondrial biogenesis and dynamics in the soleus muscle of male Wistar rats that consumed HFD.

The novelty of the study goes beyond the direct comparison of HIIT and HFD on mitochondrial function. While previous research has examined the benefits of HIIT on mitochondrial health and the detrimental effects of HFD on metabolic function, this study offers new perspectives on how these two factors can synergistically impact mitochondrial biogenesis and dynamics, exploring the balance between fission and fusion processes. The study of mitochondrial dynamics is also remarkably noteworthy. This aspect of mitochondrial function is frequently disregarded, but plays a crucial role in maintaining mitochondrial healthy and preventing dysfunction. Additionally, the use of real-time quantitative reverse transcription PCR (RT-qPCR) allows for a precise analysis of gene expression related to mitochondrial biogenesis and dynamics, providing valuable insights into the underlying mechanisms. By examining both mitochondrial biogenesis and dynamics and analyzing gene expression changes, the results of this study may provide valuable insights into the underlying mechanisms and potential clinical implications.

## Methods

### Animals and experimental design

The present study was experimental and applied. The sample consisted of twenty-four healthy male Wistar rats (age = six weeks, weight = 195 ± 15 g), purchased from Pasteur Institute in Tehran and kept in the Tarbiat Modares University animal house (Tehran, Iran). The determination of the sample size for ANOVA was achieved through the resource equation method^34^. The animals had free access to standard food and water at an environment temperature of 22 ± 1.4, a humidity of 55 ± 4, and a light-dark cycle of 12:12. After a week of familiarization with the animal house environment, the rats were randomly divided into four groups: (1) control group (Normal Diet (ND) without exercise), (2) Control + HIIT group, (3) HFD group and (4) HFD + HIIT group. Randomization was performed by the laboratory operator blindly using the numbering of rats and a lottery card. Following randomization height, weight, and Lee’s index were measured. Diet and exercise interventions started simultaneously and lasted for ten weeks. After the completion of the intervention, height, weight, and Lee’s index were measured in all groups. Experiments were conducted based on ARRIVE guidelines and the instructions of the ethics committee in research and care of animals of the Institute of Physical Education and Sports Sciences of Iran, and all stages of the study were approved by the ethics committee in research (ethics code: IR.SSRI.REC.1402.083). In addition, all measures were performed in accordance with the relevant guidelines.

### Interventions

#### Exercise training protocol

The HIIT training protocol included eight bouts of intense activity (2.5 min with an intensity of 90% of the maximum running capacity (MRC)) with periods of active rest (2.5 min with an intensity of 50% of the MRC)^35^; HIIT training was performed for ten weeks with a frequency of 3 sessions per week in the early hours of the day (light period). To familiarize and adapt to the training protocol, after a week of adaptation to the animal house environment, all rats ran on a treadmill for 10 min at a speed of 6 m per minute for one week. The MRC test - performed following group allocation - consisted of running at a speed of 6 m per minute, and gradually every 3 min, the speed increased by 3 m per minute until reaching the fatigue threshold. The inability of the rats to get to the end of the treadmill after five times of mechanical stimulation by a soft brush within 1 min is defined as the fatigue threshold speed (MRC%100), which is considered as the maximum speed and the aerobic performance of the rat. It was calculated according to the distance traveled. The rats not in the exercise training group only underwent a one-week acclimation period and the MRC test. The MRC test was repeated after ten weeks^35^.

#### Diet

During the research, rats were fed with ND and HFD prepared by the Razi Serum Institute (Iran). After the period of familiarization with the environment, the HFD and HFD + HIIT groups were fed with HFD (60% of total energy from fat derived from animal oil, 20% carbohydrates, and 20% protein) for ten weeks. Also, the control and control + HIIT groups were fed with ND (10% of total energy from fat, 70% from carbohydrates and 20% from protein) as free access.

### Body composition

A digital scale with a sensitivity of 0.01 g was used to measure the weight of the rats. The height (length) of the rats was measured using the caliper (between the tip of the nose and the beginning of the tail). The height of the rats was measured and recorded at the beginning and 48 h after the last session of the training protocol (All groups were measured at the same time of day). The weight was measured and recorded at the beginning and regularly at the end of each week until the end of the period. Lee’s index was used to evaluate the obesity status of rats. Lee’s Index is a measurement used primarily in the context of assessing obesity in rodents, particularly mice and rats. It was developed to evaluate body fatness based on the relationship between an animal’s weight and its body length ([3 square root body weight (g) / nasoanal length (cm)] *1000)^36^.

### Tissue collection

Forty-eight hours after the last training session, the rats in all groups were anesthetized by intraperitoneal injection of xylazine (10 mg/kg) and ketamine (80 mg/kg). After the injection, to confirm complete and deep anesthesia, muscle relaxation, and absence of foot and eye reflexes were checked and confirmed, then euthanasia was performed by cervical dislocation simultaneously on the same day. The soleus muscle tissue was isolated, immediately transferred into coded cryotubes, then frozen using liquid nitrogen, and immediately stored in a freezer at -80 °C. qRT-PCR method was used to investigate the expression of Pgc1-α, Mfn2, Opa1, Drp1, and Fis1 genes in soleus muscle tissue.

### Gene expression measurement (real-time PCR)

According to the manufacturer’s instructions, total RNA was extracted from soleus muscle tissues using Trizol Reagent (Kiazist, Iran). Then, the concentration of RNA samples was measured using a Nanodrop 2000 spectrophotometer (Thermo Fisher Scientific, San Jose, CA, USA). RNA was reverse transcribed using the RevertAid™ First Strand cDNA Synthesis Kit for cDNA synthesis for qPCR using SYBR Green PCR Master Mix in StepOnePlus™ (Applied Biosystems, Carlsbad, CA, USA). PCR reactions were performed under the following conditions: 15 min at 95 °C for initial denaturation, followed by 40 cycles of 15 s at 95 °C for amplification, 15 s at 95 °C and 1 min at 65 °C for the melting curve, and finally, 20 s at 30 °C for cooling. The mRNA expression content of the desired genes was calculated by standard GAPDH expression and then by 2^− CT∆∆^ method^18^. The primers used for qRT-PCR are presented in Table 1.

**Table 1:** The sequence of the forward-reverse primers of the genes desired for the real-time PCR reaction.

Gene	Forward (5′−3′)	Reverse (5′−3′)
**GAPDH**	AGGTCGGTGTGAACGGATTTG	TGTAGACCATGTAGTTGAGGTCA
**Pgc1-α**	CTTCGCTGTCATCAAACAGG	AACAAGCACTTCGGTCATCC
**Mfn2**	GAATCGGCACAGAGGAGAC	GACGGTGACGATGGAGTTG
**Opa1**	GATCATCTGCCACGGGTTGTT	GCCTTCACTGAGAGTCACCTT
**Drp1**	TTCTGAGCTATGTGGTGGT	AAATAAAGCTGGACGGGGG
**Fis1**	GGTTGCGTGGTAAGGGATG	CACCGCAGCCAGGACATAG

### Biochemical analysis

Lipid profile including Total cholesterol (TC), Total triglyceride (TG), Low-Density Lipoprotein-cholesterol (LDL-C), High-Density Lipoprotein-cholesterol (HDL-C), Very Low-Density Lipoprotein-cholesterol (VLDL-C) was measured using Abcam commercial kits (USA) by an autoanalyzer (Hettich mikro-220R, Germany).

### Statistical analysis

The Shapiro-Wilk test was used to determine the normal distribution of the data, and the Levene test was used to check the homogeneity of variance between the study groups. One-way ANOVA and Fisher’s or LSD post-hoc test were used to investigate the differences between groups. Also, the dependent T-test was used to examine intra-group changes in body weight and Lee’s index variables between the pre-test and post-test. Significance was accepted at *p* < 0.05. All data are presented as the mean ± standard deviation (SD). SPSS version 27 was used for statistical data analyses, and Excel was used to draw graphs.

## Results

Homogeneity of variance was maintained in all groups (*P* > 0.05). Also, the distribution of data in all outcomes was normal (*P* > 0.05). Serum biochemical parameters of animals are presented in Table 2.

**Table 2:** The serum characteristics lipid profile of the rats at the end of the ten weeks.

Variables	Control	Control + HIIT	HFD	HFD + HIIT
**TC**	87.8 ± 3.3	87.7 ± 3.8	177.5 ± 3.7 ‡	31.7 ± 3.9 §
**TG**	93.5 ± 6.9	96.7 ± 6.2	224 ± 5.1 ‡	212 ± 4
**LDL-C**	37.5 ± 2.8	38.4 ± 3.2	86.7 ± 3.2 ‡	57.3 ± 1.3 §
**HDL-C**	6.4 ± 1.1	6.6 ± 0.8	2.2 ± 0.5 ‡	4.6 ± 0.9 §
**VLDL-C**	17.6 ± 0.7	17.5 ± 0.8	35.5 ± 0.7 ‡	26.3 ± 0.8 §

‡ Significant difference between HFD and Control groups (*P* ≤ 0.05); § Significant difference between HFD and HFD + HIIT groups (*P* ≤ 0.05). *HIIT*: High-Intensity Interval Training; *HFD*: High-Fat Diet.

### Body weight

**Fig. 1:**
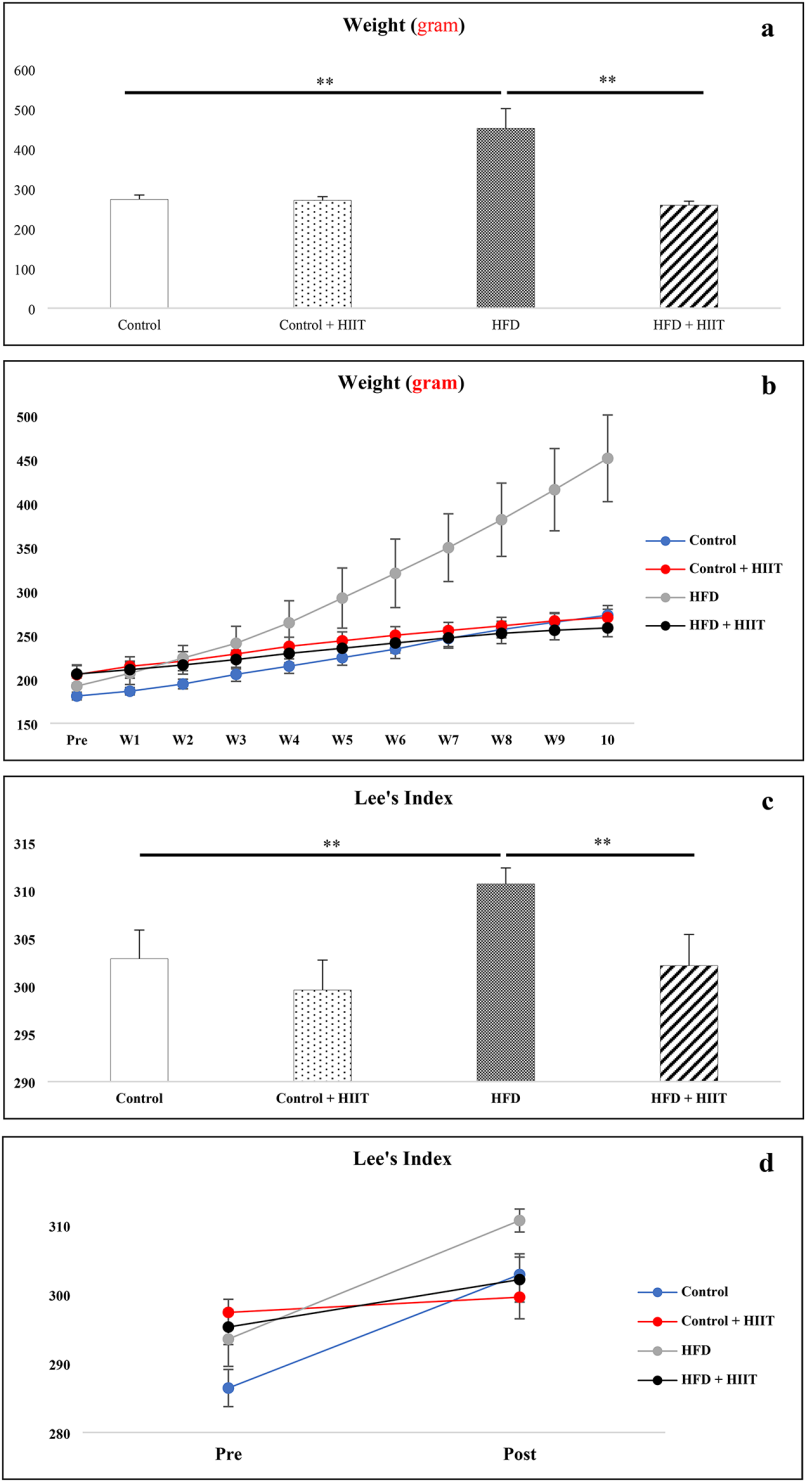
Weight and Lee’s Index Changes. HIIT training moderates HFD-induced weight gain and Lee’s index. a and b: Intergroup and Intragroup changes in weight. c and d: Intergroup and Intragroup changes of Lee’s index. *HIIT*: High-Intensity Interval Training; *HFD*: High-Fat Diet; *W*: Week. *P < 0.05; **P < 0.001.

Figure 1 shows the changes in weight (a and b) and Lee’s index (c and d) through ten weeks of intervention. At the baseline, there was no significant difference between the groups (*P* ≥ 0.05) (Table 3). After 10 weeks, the weight increased significantly in all groups (*P* < 0.001). In addition, the Lee’s index increased in all groups (*P* < 0.05), except for the control + HIIT group (*P* = 0.33). HFD consumption significantly increased body weight and Lee’s index compared to control and control + HIIT groups (*P* < 0.001). In addition, HIIT significantly reduced the weight and Lee’s index of the HFD + HIIT group compared to the HFD group (*P* < 0.001).

**Table 3:** Inter- and intra-group changes in body weight and Lee’s index variables.

Variables	Control	Control + HIIT	HFD	HFD + HIIT
**Body weight**				
*Pre*	181 ± 4.33	205.33 ± 10.23	192.33 ± 9.24	206 ± 10.46
*Post*	273 ± 10.99 †	270.33 ± 9.43 †	451.5 ± 49.32 †‡	258.5 ± 10.07 †§
**Lee’s index**				
*Pre*	286.42 ± 2.68	297.37 ± 1.89	293.52 ± 3.95	295.25 ± 2.53
*Post*	302.85 ± 3 †	299.58 ± 58	310.68 ± 1.66 †‡	302.13 ± 3.25 †§

† Significant difference between Pre and Post (*P* ≤ 0.001); ‡ Significant difference between HFD and Control groups (*P* ≤ 0.001); § Significant difference between HFD and HFD + HIIT groups (*P* ≤ 0.001). *HIIT*: High-Intensity Interval Training; *HFD*: High-Fat Diet.

### Mitochondrial biogenesis

#### Pgc1-α gene

**Fig. 2:**
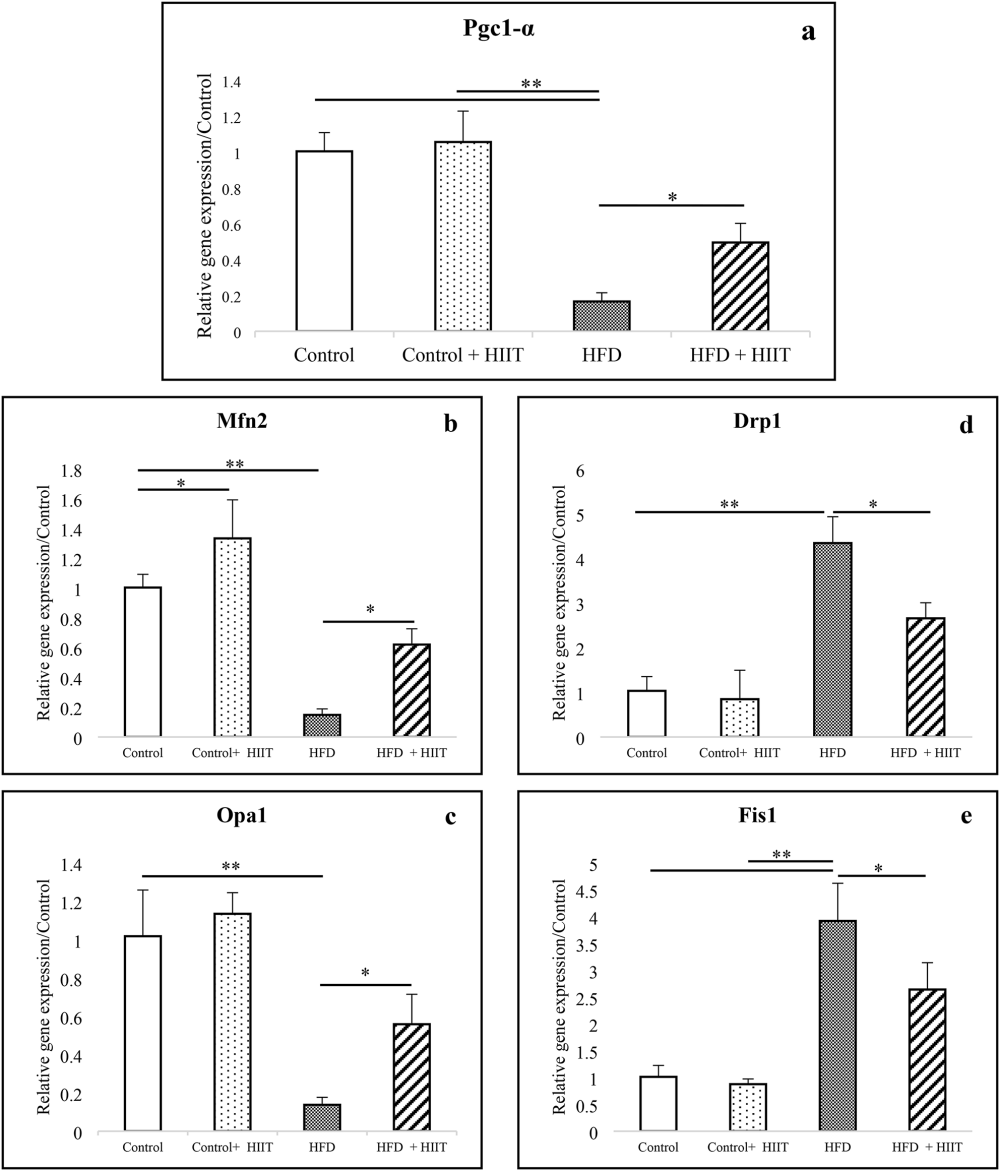
Effect of HFD consumption and HIIT exercise training on gene expression of Pgc1-α (a), Mfn2 (b), Opa1 (c), Drp1 (d), and FIS1 (e). *HIIT*: High-Intensity Interval Training; *HFD*: High-Fat Diet; *Pgc-1α*: peroxisome proliferator-activated receptor gamma coactivator 1-alpha; *Mfn2*; Mitofusins-2; *Opa1*; Optic atrophy-1; *Drp1*; Dynamin-related protein-1; *Fis1*: mitochondrial Fission protein-1. *P < 0.05; **P < 0.001.

There was a statistically significant difference between groups as determined by ANOVA (F(3, 8) = 40.004, *P* < 0.001, ES = 0.938). HFD consumption significantly decreased Pgc1-α mRNA compared to control and control + HIIT groups (*P* < 0.001). No significant difference was observed between control + HIIT and control groups (*P* = 0.6). In addition, HIIT significantly increased the Pgc1-α mRNA expression in the HFD + HIIT group compared to the HFD group (*P* = 0.009) (Fig. 2a).

### Mitochondrial dynamics

#### Mitochondrial fusion genes

##### Mfn2 and Opa1

The difference between the groups in the mRNA of Mfn2 (F(3, 8) = 35.615, *P* < 0.001, ES = 0.930) and Opa1 (F(3, 8) = 26.026, *P* < 0.001, ES = 0.907) was significant. HFD consumption decreased Mfn2 and Opa1 mRNA compared to control and control + HIIT groups (for both *P* < 0.001). HIIT increased Mfn2 gene expression in the control + HIIT group compared to the control group (*P* = 0.026). However, it was not significant in the case of Opa1 mRNA (*P* = 0.38). In addition, HIIT increased Mfn2 (*P* = 0.004) and Opa1 (*P* = 0.011) mRNA expression in the HFD + HIIT group compared to the HFD group (Fig. 2b-c).

#### Mitochondrial fission genes

##### Drp1 and Fis1

There was a difference in the expression of Drp1 (F(3, 8) = 32.230, *P* < 0.001, ES = 0.924) and Fis1 (F(3, 8) = 48.992, *P* < 0.001, ES = 0.948) genes in the groups. HFD consumption increased Drp1 and Fis1 mRNA compared to control and control + HIIT groups (for both *P* < 0.001). There was no difference between the control + HIIT group and the control group for Drp1 (*P* = 0.658) and Fis1 (*P* = 0.384). In addition, HIIT decreased Drp1 (*P* = 0.003) and Fis1 (*P* = 0.027) mRNA expression in the HFD + HIIT group compared to the HFD group (Fig. 2d-e).

## Discussion

The present study aimed to investigate the effect of HIIT on the expression of Pgc1-α, Mfn2, Opa1, Drp1 and Fis1 genes in the soleus muscle of male Wistar rats consuming HFD. Overall, the results showed that HIIT training moderates the effects of HFD consumption on related genes of mitochondrial biogenesis and dynamics.

The findings of our study indicate that HFD led to a significant increase in TC, TG, LDL-C, and VLDL-C, and a decrease in HDL-C. Furthermore, HIIT moderated this increase but did not restore the lipid profile to the values of the control group. The findings align with previous studies, Arunwan Udomkasemsab et al. (2018) showed that rats fed with HFD for 4 weeks experienced a significant increase in body weight, TC, TG, and LDL-C, while HDL-C levels decreased^37^.

Studies have shown that HIIT moderates the negative effects of HFD consumption on lipid profile^38,39^. Researchers showed that 20 weeks of HFD consumption (30% fat) increased TC and TG levels^39^. In this study, one week (4 sessions) of HIIT led to a decrease in TC and TG levels^39^. Another study also showed that 12 weeks of HFD increased the levels of TG, TC, LDL and VLDL and decreased HDL^38^. Eight weeks of HIIT and moderate-intensity continuous training (MICT) could reverse the results^38^. In addition, the results of this study showed that there was no significant difference between HIIT and MICT in reducing TG, TC, LDL, and VLDL levels. However, HIIT was significantly more effective in increasing HDL^38^.

Reduced mitochondrial biosynthesis and disruption of mitochondrial dynamics lead to mitochondrial dysfunction^40^. The Pgc-1 family (includes Pgc1-α and Pgc1-β), and peroxisome proliferator-activated receptor gamma (Ppar-γ), regulates mitochondrial biosynthesis and biogenesis^3^. Activation of Pgc-1 family consists of transcriptional coactivators under conditions of cellular energy demands, such as cell growth, hypoxia or exercise, promotes mitochondrial remodeling and intracellular energy balance^41^.

The effects of HFD on Pgc1-α levels are conflicting in the literature. Some studies have shown that HFD leads to a decrease in Pgc1-α levels, both at the gene expression^6,42^, and protein level^43^. However, other studies have demonstrated an increase in Pgc1-α protein levels^12–14^ with HFD consumption, possibly through the Ppar-δ/Ppar-γ/Pgc1-α pathway^14^. It seems that the increase in fatty acids from HFD may not always stimulate this pathway, suggesting the need for further research using knockout models to better understand the signaling pathways affected by HFD-induced increase in fatty acids.

In addition, the results showed that ten weeks of HIIT increased the expression of the Pgc1-α gene, which can help mitigate the effects of an HFD. Previous studies have also demonstrated that HIIT can increase the Pgc1-α gene and protein in various tissues, including the liver tissue^6^, and gastrocnemius muscle of diabetic male C57BL/6J mice^33^. In contrast, ten weeks of HIIT and endurance training had no significant effect on Pgc1-α protein levels in the quadriceps muscle of HFD-fed male C57BL/6J mice^35^. The quadriceps muscle is generally composed of type two fibers^44^. The density of mitochondria in type II fibers is lower than in type I. Therefore, it seems likely that Pgc1-α activity and responsiveness are low in fast-twitch muscles. While activity/expression may be low, fast-twitch muscles are actually more prone to an increase in PGC-1α expression upon training compared to slow-twitch muscles, which already have elevated basal expression. Additionally, detecting PGC-1α protein levels is notoriously challenging due to the widespread availability of nonspecific antibodies, complicating the interpretation of such results. Moreover, exercise-induced PGC-1α expression is typically transient, usually observed within 3–8 h post-exercise. This should be considered when addressing chronic elevations (i.e., > 24 h after the last exercise session, as in your study) of PGC-1α expression after a period of exercise training. As shown here, the control + HIIT group did not display increased PGC-1α expression compared to sedentary controls. However, the response of Pgc1-α to exercise training may vary depending on the type of muscle fibers and the intensity of the training. Overall, the findings suggest that HIIT training can positively impact Pgc1-α expression and may have beneficial effects on metabolic health in the context of HFD consumption.

Although the molecular mechanisms involved in the induction of mitochondrial signaling pathways by HIIT are unclear, there is evidence that intermittent effort and repetitive metabolic fluctuations in skeletal muscle may play a role^45^. Following repeated muscle contractions, changes in the concentration of several metabolites in the cytosol, including an increase in the ADP/ATP ratio, calcium flux, an increase in the NAD^+^/NADH ratio, ROS production, and redox status, regulate the expression of genes encoding proteins involved in mitochondrial biogenesis^46^. The metabolic sensors AMP-activated protein kinase (AMPK) and p38 mitogen-activated protein kinase (p38MAPK) phosphorylate Pgc1-α and regulate its transcriptional activity in skeletal muscle cells after high-intensity exercise^47,48^. Furthermore, AMPK indirectly increases Pgc1 activity in response to HIIT via SIRT-1^49,50^. However, the mechanisms of mitochondrial biosynthesis induced by the combination of exercise with HFD do not overlap^51^. Hence, more studies are needed to clarify and separate diet and exercise signaling pathways on mitochondrial biogenesis.

Phosphorylation of Ser616 and dephosphorylation of Ser637 induces Drp1 and leads to increased interaction between Drp1 and Fis1^52^. The result of this signaling pathway increases mitochondrial fission^52^. The results of the present study showed that consuming ten weeks of HFD led to a decrease in the expression of Mfn2 and Opa1 genes and a significant increase in Drp1 and Fis1. Previous studies support these results. Four weeks of HFD consumption has been shown to decrease the expression of Mfn1 and Mfn2 and increase Fis1 and Drp1^53^. Other studies have shown differences based on the diet type: saturated fat increased Drp1 and FIS1, while unsaturated fat increased Mfn1 and Mfn2, leading to improved mitochondrial function^54,55^. Recently, researchers have reported that 24 weeks of HFD consumption leads to a significant decrease in the levels of Mfn2 and Opa1 proteins in various tissues, including the heart, liver, spleen, kidneys, and quadriceps muscle of C57bL6j male mice^56^. The answer to whether changes in gene expression are harmful is not straightforward. However, changes in gene expression can be associated with changes in protein levels^10,11^. It seems, that regarding changes and reduction in gene expression related to mitochondrial biogenesis and dynamics, the impact on an organism’s fitness and health depends on various factors, such as the specific genes involved, the type of change, and the organism’s environment. Hence, it seems that HFD may have harmful consequences on muscles and even other body tissues.

Also, Drp1 protein levels following HFD consumption in different tissues showed different results. So that Drp1 decreased in the heart and liver, and increased in the kidney and quadriceps muscles^56^. It seems that the functional nature of the tissues has an important role in their influence from diet. Mitochondrial distribution is strongly associated with high energy demand^57^.

The results of the present study showed that HIIT for ten weeks increased the expression of Mfn2 and Opa1 genes, while decreasing the expression of Drp1 and Fis1 genes. This is consistent with previous studies in the liver^6^ and soleus muscle^29^ in diabetic mice, where HIIT training was found to improve mitochondrial dynamics by increasing Mfn2 mRNA and decreasing Drp1 gene expression. In another study, the positive effect of exercise training on mitochondrial dynamics in the brain has also been shown. Eight weeks of continuous progressive aerobic training increased the levels of fusion (Mfn1, Mfn2, and Opa1) and decreased fission (Drp1 and Fis1) proteins in the hippocampus of male Sprague-Dawley rats fed an HFD for 16 weeks^31^. These results show that exercise moderates the consequences of HFD consumption on mitochondrial dynamics. On the other hand, various human studies have reported that endurance^58,59^, HIIT^58,60^, and Sprint Interval Training (SIT)^58–60^ induce mitochondrial fusion in healthy subjects by increasing Mfn2 gene expression^59^ and protein levels^58,60^. Exercise training induces the phosphorylation of Drp1 at Ser637 through AMPK-mediated activation of A-kinase anchor protein 1 (AKAP1) and inhibits the interaction of Drp1 and Fis1, thereby inhibiting mitochondrial fission^52^. In addition, exercise training affects the glutathione antioxidant (GSH) system by increasing the use of oxygen during exercise and leads to an increase in GSH oxidation. Also, exercise training by increasing the production of oxidized glutathione (GSSG) and increasing the GSSG/GSH ratio leads to disulfide (-S-S-)-mediated Mfn oligomers and then induces mitochondrial fusion^52^.

On the contrary, it has been demonstrated that six weeks of HFD (45%) increased the levels of Mfn2, Drp1, and Fis1 proteins^12^, which is contradictory to the results of the previous study^6,33^. Because Pgc1-α has been shown to induce the expression of Mfn2^15^. It seems that the cause of Mfn2 increase is the induction of Pgc1-α by increasing fatty acids. In addition, the muscle response to low-intensity endurance or HIIT training significantly increases the levels of Mfn1, Mfn2, and Drp1 proteins, while significantly decreasing Fis1 levels in the EDL muscle of male Sprague-Dawley rats^12^ and gastrocnemius muscle of C57BL/6J diabetic male mice^33^. The type of fat in the HFD (saturated or unsaturated fat), access to food, target tissue (fast twitch or slow twitch muscles, liver, brain, etc.), and exercise protocol are factors that may contribute to the differences in results observed in different studies. Overall, the molecular and cellular responses of tissues to HFD and exercise can vary, suggesting a complex interplay between diet, exercise, and mitochondrial dynamics proteins.

### Limitations and future research directions

In the present study, only gene expression was analyzed. Considering that the increase in gene expression is not necessarily associated with the increase in protein, it has been shown that Pgc1-α protein levels are increased under the influence of HFD through the increase and activation of Pparδ without increasing its gene expression^14^. Therefore, it is recommended to investigate gene expression and protein levels involved in the biogenesis up-and-down-regulation of the signaling pathway. Another limitation of this study and previous studies is the lack of measurement of calories consumed. Therefore, it is suggested that future studies in this area measure the amount of calories consumed. Due to the lack of assessment of changes in the functional indicators of mitochondria and morphology, it is suggested that future studies examine the number and size of mitochondria using an electron microscope. It is also recommended to check the variables of mitochondrial respiration.

The results of this study may contribute to our understanding of the complex interaction between exercise, nutrition and mitochondrial health and pave the way for future research in this area. Although this study provides important insights into the short-term effects of HIIT and HFD on mitochondrial function, more research is needed to examine the long-term effects of these exercises. Understanding the long-term effects of HIIT and HFD on mitochondrial health is important in developing disease prevention and treatment strategies. Additionally, the study focused on male Wistar rats. Future research should investigate whether the results can be generalized to other populations, including humans and individuals with different genetic backgrounds or health status. Understanding individual variability in response to HIIT and HFD is important to tailor interventions to specific needs.

### Implications

The study’s findings could have important practical implications for individuals looking to enhance their health through physical activity and diet. A better comprehension of how HIIT and HFD interact to affect mitochondrial function can enable healthcare providers to offer more personalized advice on exercise and nutrition. Furthermore, mitochondrial dysfunction is linked to a range of conditions, such as metabolic disorders and neurodegenerative diseases. The results of the study suggest that HIIT could be a promising approach to preventing or alleviating these conditions by preserving mitochondrial health.

Moreover, the results of this study demonstrate the importance of considering both mitochondrial biogenesis and dynamics when examining the effects of exercise and diet on mitochondrial health. Furthermore, the study’s focus on gene expression provides valuable insight into the underlying mechanisms by which HIIT may counteract the detrimental effects of HFD on mitochondrial function. By identifying specific genes involved in mitochondrial biogenesis and dynamics, the results of this study contribute to our understanding of the molecular pathways involved.

This study’s key strength lies in the combined application of HFD regimen and HIIT interventions, which showed promising results in preventing and managing the consequences of HFD. While the findings may not directly translate from animal models to humans, they suggest that individuals consuming an HFD can benefit from HIIT to mitigate its consequences. This study underscores the potential of HIIT as a preventive measure for weight management and obesity, offering valuable insights for healthcare professionals and coaches. Future research should prioritize human subjects to validate and generalize these results.

## Conclusion

Based on the results, HFD and HIIT have opposite effects on genes related to mitochondrial biogenesis and dynamics. Thus, HFD consumption reduces the expression of genes related to mitochondrial biogenesis and fusion. HIIT increases the expression of biogenesis and fusion genes. Also, HIIT moderated the increase in the expression of mitochondrial fission genes caused by HFD. As well as, HIIT training as a non-pharmacological treatment can manage weight and obesity status.

## Data Availability

All data generated or analyzed during this study are included in this published article.

## Abbreviations

AKAP1: AMPK-mediated activation of A-kinase anchor protein 1
AMPK: AMP-activated protein kinase
DRP1: Dynamin-related protein-1
EDL: Extensor digitorum longus
FIS1: Mitochondrial fission protein-1
HDL-C: High-density lipoprotein-cholesterol
HFD: High-fat diet
HIIT: High-intensity interval training
LDL-C: Low-density lipoprotein-cholesterol
MFN2: Mitofusins-2
MRC: Maximum running capacity
ND: Normal diet
Opa1: Optic atrophy-1
p38MAPK: p38 mitogen-activated protein kinase
PGC-1α: Peroxisome proliferator-activated receptor gamma coactivator-1-alpha
PPAR-γ: Peroxisome proliferator-activated receptor gamma
TC: Total cholesterol
TG: Total triglyceride
VLDL-C: Very low-density lipoprotein-cholesterol

## References

[1] W. H. Organization. *World Health Organization Obesity and Overweight.* (2024). https://www.who.int/news-room/fact-sheets/detail/obesity-and-overweight. Accessed 1 Mar 2024.

[2] Cojocaru, K. A. et al. Mitochondrial dysfunction, oxidative stress, and therapeutic strategies in diabetes, obesity, and cardiovascular disease. *Antioxidants* **12**, 658. 10.3390/antiox12030658 (2023). 36978905 10.3390/antiox12030658 PMC10045078

[3] Gu, C., Yan, J., Zhao, L., Wu, G. & Wang, Y. Regulation of mitochondrial dynamics by aerobic exercise in cardiovascular diseases. *Front. Cardiovasc. Med.* **8**, 2001. 10.3389/fcvm.2021.788505 (2022). 10.3389/fcvm.2021.788505 PMC8793839 35097008

[4] Giulivi, C., Zhang, K. & Arakawa, H. Recent advances and new perspectives in mitochondrial dysfunction. *Sci. Rep.* **13**, 7977. 10.1038/s41598-023-34624-8 (2023). 37198256 10.1038/s41598-023-34624-8 PMC10192368

[5] Endalifer, M. L., Diress, G. & Epidemiology predisposing factors, biomarkers, and prevention mechanism of obesity: A systematic review. *J Obes.* 6134362 (2020). 10.1155/2020/6134362 10.1155/2020/6134362 PMC7281819 32566274

[6] Wang, Y. et al. HIIT ameliorates inflammation and lipid metabolism by regulating macrophage polarization and mitochondrial dynamics in the liver of type 2 diabetes mellitus mice. *Metabolites* **13**, 14. 10.3390/metabo13010014 (2022). 36676939 10.3390/metabo13010014 PMC9862084

[7] Srisutha, J. et al. P2X7R and P2X4R expression of mice submandibular gland in high-fat diet/streptozotocin-induced type 2 diabetes. *Sci. Rep.* **14**, 10855. 10.1038/s41598-024-60519-3 (2024). 38740782 10.1038/s41598-024-60519-3 PMC11091137

[8] Sun, J. et al. Early mitochondrial adaptations in skeletal muscle to obesity and obesity resistance differentially regulated by high-fat diet. *Exp. Clin. Endocrinol. Diabetes*. **125**, 538–546. 10.1055/s-0043-104634 (2017). 28444662 10.1055/s-0043-104634

[9] Kyriazis, I. D. et al. The impact of diet upon mitochondrial physiology. *Int. J. Mol. Med.* **50**, 1–26. 10.3892/ijmm.2022.5191 (2022). 10.3892/ijmm.2022.5191 PMC9542544 36129147

[10] Sparks, L. M. et al. A high-fat diet coordinately downregulates genes required for mitochondrial oxidative phosphorylation in skeletal muscle. *Diabetes* **54**, 1926–1933. 10.2337/diabetes.54.7.1926 (2005). 15983191 10.2337/diabetes.54.7.1926

[11] Kang, K. W. et al. Diastolic dysfunction induced by a high-fat diet is associated with mitochondrial abnormality and adenosine triphosphate levels in rats. *Endocrinol. Metab. (Seoul)*. **30**, 557–568. 10.3803/enm.2015.30.4.557 (2015). 26790384 10.3803/EnM.2015.30.4.557 PMC4722412

[12] Kang, Y. S., Seong, D., Kim, J. C. & Kim, S. H. Low-intensity exercise training additionally increases mitochondrial dynamics caused by high-fat diet (HFD) but has no additional effect on mitochondrial biogenesis in fast-twitch muscle by HFD. *Int. J. Environ. Res. Public. Health*. **17**, 5461. 10.3390/ijerph17155461 (2020). 32751208 10.3390/ijerph17155461 PMC7432492

[13] Fillmore, N., Jacobs, D. L., Mills, D. B., Winder, W. W. & Hancock, C. R. Chronic AMP-activated protein kinase activation and a high-fat diet have an additive effect on mitochondria in rat skeletal muscle. *J. Appl. Physiol.* **109**, 511–520. 10.1152/japplphysiol.00126.2010 (2010). 20522731 10.1152/japplphysiol.00126.2010 PMC2928588

[14] Hancock, C. R. et al. High-fat diets cause insulin resistance despite an increase in muscle mitochondria. *Proc. Natl. Acad. Sci. U S A*. **105**, 7815–7820. 10.1073/pnas.0802057105 (2008). 18509063 10.1073/pnas.0802057105 PMC2409421

[15] Green, A., Hossain, T. & Eckmann, D. M. Mitochondrial dynamics involves molecular and mechanical events in motility, fusion and fission. *Front. Cell. Dev. Biol.* **10**, 1010232. 10.3389/fcell.2022.1010232 (2022). 36340034 10.3389/fcell.2022.1010232 PMC9626967

[16] Liu, R. et al. Impaired mitochondrial dynamics and bioenergetics in diabetic skeletal muscle. *Plos One*. **9**, e92810. 10.1371/journal.pone.0092810 (2014). https://doi.org/https://doi.org/24658162 10.1371/journal.pone.0092810 PMC3962456

[17] Jheng, H. F. et al. Mitochondrial fission contributes to mitochondrial dysfunction and insulin resistance in skeletal muscle. *Mol. Cell. Biol.* **32**, 309–319. 10.1128/mcb.05603-11 (2012). 22083962 10.1128/MCB.05603-11 PMC3255771

[18] Roy, M., Reddy, P. H., Iijima, M. & Sesaki, H. Mitochondrial division and fusion in metabolism. *Curr. Opin. Cell. Biol.* **33**, 111–118. 10.1016/j.ceb.2015.02.001 (2015). 25703628 10.1016/j.ceb.2015.02.001 PMC4380865

[19] Drake, J. C., Wilson, R. J. & Yan, Z. Molecular mechanisms for mitochondrial adaptation to exercise training in skeletal muscle. *FASEB J.* **30**, 13. 10.1096/fj.15-276337 (2016). 26370848 10.1096/fj.15-276337 PMC6137621

[20] Kim, Y., Triolo, M. & Hood, D. A. Impact of aging and exercise on mitochondrial quality control in skeletal muscle. *Oxid. Med. Cell. Longev.* **2017**10.1155/2017/3165396 (2017). 10.1155/2017/3165396 PMC5471566 28656072

[21] Lee, H. et al. A cellular mechanism of muscle memory facilitates mitochondrial remodelling following resistance training. *J. Physiol.* **596**, 4413–4426. 10.1113/jp275308 (2018). 30099751 10.1113/JP275308 PMC6138296

[22] Greene, N. P. et al. Mitochondrial quality control, promoted by PGC-1α, is dysregulated by Western diet‐induced obesity and partially restored by moderate physical activity in mice. *Physiol. Rep.* **3**, e12470. 10.14814/phy2.12470 (2015). 26177961 10.14814/phy2.12470 PMC4552545

[23] Heo, J. W. et al. Effects of exercise on obesity-induced mitochondrial dysfunction in skeletal muscle. *Korean J. Physiol. Pharmacol.* **21**, 567–577. 10.4196/kjpp.2017.21.6.567 (2017). 29200899 10.4196/kjpp.2017.21.6.567 PMC5709473

[24] Uemichi, K., Shirai, T., Hanakita, H. & Takemasa, T. Effect of mechanistic/mammalian target of rapamycin complex 1 on mitochondrial dynamics during skeletal muscle hypertrophy. *Physiol. Rep.* **9**, e14789. 10.14814/phy2.14789 (2021). 33660929 10.14814/phy2.14789 PMC7931617

[25] Kitaoka, Y., Nakazato, K. & Ogasawara, R. Combined effects of resistance training and calorie restriction on mitochondrial fusion and fission proteins in rat skeletal muscle. *J. Appl. Physiol.* **121**, 806–810. 10.1152/japplphysiol.00465.2016 (2016). 27539498 10.1152/japplphysiol.00465.2016

[26] Takegaki, J. et al. Influence of shortened recovery between resistance exercise sessions on muscle-hypertrophic effect in rat skeletal muscle. *Physiol. Rep.* **7**, e14155. 10.14814/phy2.14155 (2019). 31250976 10.14814/phy2.14155 PMC6598394

[27] Chavanelle, V. et al. Effects of high-intensity interval training and moderate-intensity continuous training on glycaemic control and skeletal muscle mitochondrial function in db/db mice. *Sci. Rep.* **7**, 1–10. 10.1038/s41598-017-00276-8 (2017). 28303003 10.1038/s41598-017-00276-8 PMC5427962

[28] Delfan, M. et al. Effects of two workload-matched high intensity interval training protocols on regulatory factors associated with mitochondrial biogenesis in the soleus muscle of diabetic rats. *Front. Physiol.* **1730**10.3389/fphys.2022.927969 (2022). 10.3389/fphys.2022.927969 PMC9541894 36213227

[29] Peyravi, A., Yazdanpanahi, N., Nayeri, H. & Hosseini, S. A. The effect of endurance training with crocin consumption on the levels of MFN2 and DRP1 gene expression and glucose and insulin indices in the muscle tissue of diabetic rats. *J. Food Biochem.* **44**, e13125. 10.1111/jfbc.13125 (2020). 31849103 10.1111/jfbc.13125

[30] Cho, J. et al. Exercise training attenuates pulmonary inflammation and mitochondrial dysfunction in a mouse model of high-fat high-carbohydrate-induced NAFLD. *BMC Med.* **20**, 1–12. 10.1186/s12916-022-02629-1 (2022). 36348343 10.1186/s12916-022-02629-1 PMC9644617

[31] Koo, J. H. & Kang, E. B. Effects of treadmill exercise on the regulatory mechanisms of mitochondrial dynamics and oxidative stress in the brains of high-fat diet fed rats. *J. Exerc. Nutr. Biochem.* **23**, 28. 10.20463/jenb.2019.0005 (2019). 10.20463/jenb.2019.0005 PMC6477818 31010272

[32] Heo, J. W. et al. Moderate aerobic exercise training ameliorates impairment of mitochondrial function and dynamics in skeletal muscle of high-fat diet‐induced obese mice. *FASEB J.* **35**, e21340. 10.1096/fj.202002394r (2021). 33455027 10.1096/fj.202002394R

[33] Zheng, L., Rao, Z., Guo, Y., Chen, P. & Xiao, W. High-intensity interval training restores glycolipid metabolism and mitochondrial function in skeletal muscle of mice with type 2 diabetes. *Front. Endocrinol. (Lausanne)*. **11**, 561. 10.3389/fendo.2020.00561 (2020). 32922365 10.3389/fendo.2020.00561 PMC7456954

[34] Charan, J. & Kantharia, N. How to calculate sample size in animal studies? *J. Pharmacol. Pharmacother*. **4**, 303–306. 10.4103/0976-500x.119726 (2013). 24250214 10.4103/0976-500X.119726 PMC3826013

[35] Martinez-Huenchullan, S. F. et al. Constant-moderate and high-intensity interval training have differential benefits on insulin sensitive tissues in high-fat fed mice. *Front. Physiol.* **10**, 459. 10.3389/fphys.2019.00459 (2019). 31105582 10.3389/fphys.2019.00459 PMC6494961

[36] Bernardis, L. L., Bellinger, L. L., Spinner, L. I. & Brooks, S. Feeding studies in weanling rats with dorsomedial hypothalamic lesions: Effect of high fat and high carbohydrate diet and nutrient completeness on food choice and intake. *J. Nutr.* **108**, 753–758. 10.1093/jn/108.5.753 (1978). 641591 10.1093/jn/108.5.753

[37] Udomkasemsab, A. & Prangthip, P. High fat diet for induced dyslipidemia and cardiac pathological alterations in Wistar rats compared to Sprague Dawley rats. *Clínica E Investigación en Arterioscler.* **31**, 56–62. 10.1016/j.arteri.2018.09.004 (2019). 10.1016/j.arteri.2018.09.004 30591270

[38] Rahmati-Ahmadabad, S., Azarbayjani, M. A., Farzanegi, P. & Moradi, L. High-intensity interval training has a greater effect on reverse cholesterol transport elements compared with moderate-intensity continuous training in obese male rats. *Eur. J. Prev. Cardiol.* **28**, 692–701. 10.1177/2047487319887828 (2021). 33611472 10.1177/2047487319887828

[39] de Oliveira França, G. et al. Effects of short-term high-intensity interval and continuous exercise training on body composition and cardiac function in obese sarcopenic rats. *Life Sci.* **256**, 117920. 10.1016/j.lfs.2020.117920 (2020). 32522571 10.1016/j.lfs.2020.117920

[40] Bullon, P. & Marin-Aguilar, F. Roman-Malo, L. AMPK/mitochondria in metabolic diseases. *Exp. Suppl.* 129–152. 10.1007/978-3-319-43589-3_6 (2016). 10.1007/978-3-319-43589-3_6 27812979

[41] Martin, O. J. et al. A role for peroxisome proliferator-activated receptor γ coactivator-1 in the control of mitochondrial dynamics during postnatal cardiac growth. *Circ. Res.* **114**, 626–636. 10.1161/circresaha.114.302562 (2014). 24366168 10.1161/CIRCRESAHA.114.302562 PMC4061768

[42] Koves, T. R. et al. Peroxisome proliferator-activated receptor-γ co-activator 1α-mediated metabolic remodeling of skeletal myocytes mimics exercise training and reverses lipid-induced mitochondrial inefficiency. *J. Biol. Chem.* **280**, 33588–33598. 10.1074/jbc.m507621200 (2005). 16079133 10.1074/jbc.M507621200

[43] Kawanishi, N. et al. Endurance exercise training and high-fat diet differentially affect composition of diacylglycerol molecular species in rat skeletal muscle. *Am. J. Physiol. Regul. Integr. Comp. Physiol.* **314**, R892–R901. 10.1152/ajpregu.00371.2017 (2018). 29443549 10.1152/ajpregu.00371.2017 PMC6032301

[44] Klover, P., Chen, W., Zhu, B. M. & Hennighausen, L. Skeletal muscle growth and fiber composition in mice are regulated through the transcription factors STAT5a/b: linking growth hormone to the androgen receptor. *FASEB J.* **23**, 3140. 10.1096/fj.08-128215 (2009). 19417088 10.1096/fj.08-128215 PMC2735360

[45] Combes, A. et al. Exercise-induced metabolic fluctuations influence AMPK, p38‐MAPK and ca MKII phosphorylation in human skeletal muscle. *Physiol. Rep.* **3**, e12462. 10.14814/phy2.12462 (2015). 26359238 10.14814/phy2.12462 PMC4600372

[46] MacInnis, M. J. & Gibala, M. J. Physiological adaptations to interval training and the role of exercise intensity. *J. Physiol.* **595**, 2915–2930. 10.1113/jp273196 (2017). 27748956 10.1113/JP273196 PMC5407969

[47] Gibala, M. J. et al. Brief intense interval exercise activates AMPK and p38 MAPK signaling and increases the expression of PGC-1α in human skeletal muscle. *J. Appl. Physiol.* **106**, 929–934. 10.1152/japplphysiol.90880.2008 (2009). 19112161 10.1152/japplphysiol.90880.2008

[48] Bartlett, J. D. et al. Matched work high-intensity interval and continuous running induce similar increases in PGC-1α mRNA, AMPK, p38, and p53 phosphorylation in human skeletal muscle. *J. Appl. Physiol.* **112**, 1135–1143. 10.1152/japplphysiol.01040.2011 (2012). 22267390 10.1152/japplphysiol.01040.2011

[49] Cantó, C. & Auwerx, J. PGC-1alpha, SIRT1 and AMPK, an energy sensing network that controls energy expenditure. *Curr. Opin. Lipidol.* **20**, 98. 10.1097/mol.0b013e328328d0a4 (2009). 19276888 10.1097/MOL.0b013e328328d0a4 PMC3627054

[50] Gurd, B. J., Perry, C. G., Heigenhauser, G. J., Spriet, L. L. & Bonen, A. High-intensity interval training increases SIRT1 activity in human skeletal muscle. *Appl. Physiol. Nutr. Metab.* **35**, 350–357. 10.1139/h10-030 (2010). 20555380 10.1139/H10-030

[51] Koh, J. H. et al. PPARβ is essential for maintaining normal levels of PGC-1α and mitochondria and for the increase in muscle mitochondria induced by exercise. *Cell Metab.* **25**, 1176–1185. e1175 (2017). 10.1016/j.cmet.2017.04.029 10.1016/j.cmet.2017.04.029 PMC5894349 28467933

[52] Tanaka, T. et al. Mitochondrial dynamics in exercise physiology. *Pflugers Arch.* **472**, 137–153. 10.1007/s00424-019-02258-3 (2020). 30707289 10.1007/s00424-019-02258-3

[53] Cerveny, K. L., Tamura, Y., Zhang, Z., Jensen, R. E. & Sesaki, H. Regulation of mitochondrial fusion and division. *Trends Cell. Biol.* **17**, 563–569. 10.1016/j.tcb.2007.08.006 (2007). 17959383 10.1016/j.tcb.2007.08.006

[54] Putti, R., Migliaccio, V., Sica, R. & Lionetti, L. Skeletal muscle mitochondrial bioenergetics and morphology in high fat diet induced obesity and insulin resistance: Focus on dietary fat source. *Front. Physiol.* **6**, 426. 10.3389/fphys.2015.00426 (2016). 26834644 10.3389/fphys.2015.00426 PMC4719079

[55] Putti, R., Sica, R., Migliaccio, V. & Lionetti, L. Diet impact on mitochondrial bioenergetics and dynamics. *Front. Physiol.* **6**, 109. 10.3389/fphys.2015.00109 (2015). 25904870 10.3389/fphys.2015.00109 PMC4389347

[56] Zheng, P. et al. High-fat diet causes mitochondrial damage and downregulation of mitofusin-2 and optic atrophy-1 in multiple organs. *J. Clin. Biochem. Nutr.* **73**, 61. 10.3164/jcbn.22-73 (2023). 37534099 10.3164/jcbn.22-73 PMC10390808

[57] Picard, M., White, K. & Turnbull, D. M. Mitochondrial morphology, topology, and membrane interactions in skeletal muscle: A quantitative three-dimensional electron microscopy study. *J. Appl. Physiol.* **114**, 161–171. 10.1152/japplphysiol.01096.2012 (2013). 23104694 10.1152/japplphysiol.01096.2012 PMC3544498

[58] Granata, C., Oliveira, R. S., Little, J. P., Renner, K. & Bishop, D. J. Training intensity modulates changes in PGC-1α and p53 protein content and mitochondrial respiration, but not markers of mitochondrial content in human skeletal muscle. *FASEB J.* **30**, 959–970. 10.1096/fj.15-276907 (2016). 26572168 10.1096/fj.15-276907

[59] Fiorenza, M. et al. Metabolic stress-dependent regulation of the mitochondrial biogenic molecular response to high‐intensity exercise in human skeletal muscle. *J. Physiol.* **596**, 2823–2840. 10.1113/jp275972 (2018). 29727016 10.1113/JP275972 PMC6046074

[60] Huertas, J. R. et al. Human muscular mitochondrial fusion in athletes during exercise. *FASEB J.* **33**, 12087–12098. 10.1096/fj.201900365rr (2019). 31398297 10.1096/fj.201900365RR

